# The role of surface reduction in the formation of Ti interstitials†

**DOI:** 10.1039/c9ra01015g

**Published:** 2019-04-17

**Authors:** Julian Gaberle, Alexander Shluger

**Affiliations:** Department of Physics and Astronomy, University College London Gower Street WC1E 6BT London UK a.shluger@ucl.ac.uk

## Abstract

Density functional theory simulations are used to investigate the formation and mobility of Ti interstitial ions, Ti_i_, at the (110) surface of rutile TiO_2_. Interstitials were found to be favoured in the second layer below the surface plane, where they induce electron polaron states at surface and subsurface lattice Ti atoms. Reduction of the surface significantly lowers the barrier for Ti_i_ formation at the surface: the barrier for formation of Ti_i_ is reduced to just ∼0.5 eV for a Ti atom next to two bridging oxygen vacancies. However, the barrier to separate the interstitial from the surface oxygen vacancies is ∼2.5 eV. The bulk diffusion barrier is recovered after the interstitial is moved away from the vacancy complex. These results support an experimentally postulated mechanism of Ti_i_ formation and contribute to our understanding of the TiO_2_ surface reduction and reoxidation.

## Abstract 2

Density functional theory simulations are used to investigate the formation and mobility of Ti interstitial ions, Ti_i_, at the (110) surface of rutile TiO_2_.

## Graphical abstract

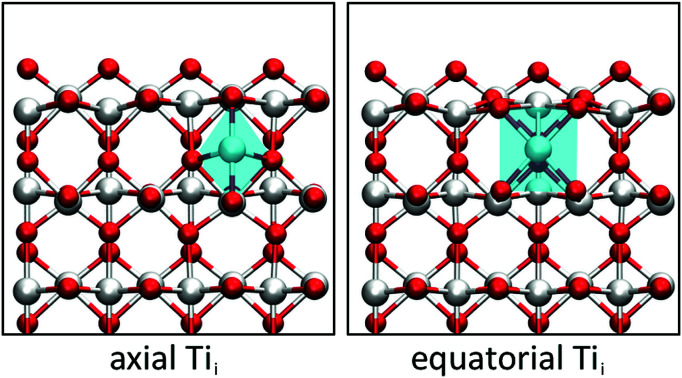

## 1 Introduction

Titanium dioxide (TiO_2_) finds applications in many different areas, such as paints and coatings,^1,2^ catalysis,^3,4^ optical instruments,^5^ solar cells^6,7^ and gas sensors.^8,9^ Its high refractive index is exploited in sunscreen and to make white pigments. The discovery of the ability of TiO_2_ to split water for hydrogen production has fuelled intense research.^10–13^ TiO_2_ displays rich defect chemistry and in fact most applications of TiO_2_ exploit or rely on such defects. For example in solar cell technologies reduced or hydrogenated TiO_2_ displays enhanced photo-absorption in the visible and IR spectral region.^14,15^

During the preparation of TiO_2_ samples defects can easily be introduced, which results in high conductivity and enhanced catalytic activity.^16^ Indeed, rutile TiO_2_ has a very rich phase diagram with many substoichiometric phases of the type Ti_*n*_O_2*n*−1_, termed Magneli phases, which are related to the formation of crystallographic shear planes.^16^ In the range of TiO_1.9996_ to TiO_1.9999_ (3.7 × 10^18^ to 1.3 × 10^19^ missing O atoms per cm^3^) interstitial Ti atoms are the dominant defects.^17^ Oxygen can be removed through high temperature annealing or ion sputtering, which are commonly used procedures in sample preparation for surface probe experiments.^18,19^ Consequently, many different surface structures of varying defect density and reconstructions have been reported.^16,20,21^ It is commonly accepted that excess Ti in the form of interstitial atoms results from oxygen removal, which is a surface mediated effect. However, the Ti defects can penetrate the entire crystal.^22,23^

The experimental evidence for Ti^3+^ species, which are linked to Ti interstitials (Ti_i_) as well as oxygen vacancies (v_O_), is considerable: the introduction of Ti_i_ leads to the crystal changing colour from transparent to blue with increasing Ti_i_ concentration, which is related to d–d transitions.^24^ Further, a gap state about 1 eV below the CBM is induced as measured by photo-electron spectroscopy and energy loss experiments.^25–27^ A Ti 3d^1^ state is also responsible for the measured ***g***-tensor in EPR measurements^28,29^ and Ti-3d states are observed in Auger electron spectroscopy.^30^

In isotopically labelled secondary ion mass spectrometry experiments, Ti interstitials were identified to be the main diffusing species at temperatures above 400 K.^22^ However, at temperatures below 800 K the concentration of surface Ti_i_ accounts for more than 95% compared to bulk. The balance starts to shift with increasing temperature with a more even split at 1073 K annealing temperature indicating indiffusion of Ti_i_ species.^31^

A series of STM measurements during a sample anneal at 1000 K show shrinking step edges on the (110) surface within a few minutes. Longer annealing leads to the formation of oxygen vacancies, v_O_, on the surface and ultimately to the cross-linked 1 × 2 reconstruction. It is postulated that oxygen is removed from the crystal into the gas phase and Ti moves as an interstitial into the bulk crystal.^32^ The exact atomic structure of the cross-linked reconstruction is still contested,^33–36^ yet proposed models all show strands of missing Ti and O atoms along the [001] direction.^18,34^ STM studies of lightly reduced rutile TiO_2_ (110) surface show isolated oxygen vacancies.^16,21,32,37,38^ To form the cross-linked reconstruction vacancies must coalesce, but the exact mechanism of this process is yet unclear. In this paper, we examine whether the formation of two neighbouring bridging oxygen vacancies can facilitate the formation of Ti interstitials and their diffusion into the bulk crystal.

The inverse of this process has also been studied experimentally: reoxidation studies show that surface adsorbed oxygen from the gas phase can react with Ti_i_ from the bulk to grow new strands,^32,39^ islands^23^ and ultimately complete 1 × 1 layers of TiO_2_,^16,32,38^ indicating that the bulk crystal behaves as a defect reservoir. The uptake rate of O_2_ is dependent on the reduction state of the sample, with faster uptake for the more reduced samples.^40^

Surface reoxidation of reduced crystals is a common procedure for STM experiments, since a conducting bulk reduced crystal is needed and reoxidising the surface can yield a near defect-free 1 × 1 (110) surface.^16,40^ While the complex defect structure underneath the reoxidised surface remains hidden, it impacts island growth^38^ and is responsible in part for a dependence of crystal reduction state, colour, surface structure or surface reactivity on sample treatment and sample history.^16,32,40^

Further evidence of Ti_i_ diffusion into the bulk was reported from EPR measurements.^41^ The ratio of surface to bulk Ti^3+^ species was measured as a function of annealing temperature. It was shown that the surface signal dominates at annealing temperatures of 773 K. As the temperature is raised, the bulk signal gains in intensity, but the surface Ti^3+^ signal remains stronger at all annealing temperatures.

Theoretical studies of near surface Ti_i_ species and their indiffusion mechanism are still rare. The Ti_i_ injection barrier has recently been reported from density functional theory (DFT) GGA+U calculations^42^ and a micro-kinetic model.^43^ However, no detailed first principles calculations of the indiffusion of Ti from a reduced surface have been reported. Experimental evidence for Ti_i_ diffusion shows linear increase of diffusion coefficients with temperature for slightly reduced crystals, which breaks down for highly reduced samples.^44^ However, the reported diffusion barrier of 2.47 eV is an order of magnitude larger than bulk diffusion barriers predicted by DFT calculations,^45,46^ motivating the investigation of Ti_i_ diffusion at the pristine and reduced rutile TiO_2_ (110) surface.

In this work, DFT simulations are used to shed further light on the formation and diffusion of Ti_i_ atoms at the (110) surface. Particularly the process of formation and subsequent diffusion of the Ti_i_ away from the surface defect into the bulk is studied for stoichiometric as well as reduced (110) surfaces. The results show that the barrier for formation of Ti_i_ is reduced to just ∼0.5 eV for a Ti atom next to two bridging oxygen vacancies. In this configuration the energy of the interstitial site is lower than that of the Ti atom at its lattice site. However, the barrier to dissociate the interstitial from the defect complex is ∼2.5 eV. Ti_i_ quickly recovers a bulk diffusion barrier as it moves away from the Ti vacancy (v_Ti_) inside the sample.

## 2 Methods

The calculations for rutile TiO_2_ were performed using DFT as implemented in the CP2K code,^47^ which employs a mixed Gaussian and plane wave basis-set (GPW). A triple zeta basis set was used for Ti and O together with Goedecker–Teter–Hutter (GTH) pseudo-potentials.^48^ In order to get an improved description of the electronic and geometric structure, the Ti 3s^2^, 3p^6^, 3d^2^ and 4s^2^ electrons were treated as valence. The plane wave cutoff was converged at 600 Ry, SCF convergence was set to 10^−6^ a.u. and residual forces on relaxed atoms were converged to smaller than 0.01 eV Å^−1^. A detailed description of the setup and validation is given in ESI.†

In the generalised gradient approximation rutile is predicted to be unstable, since imaginary phonon frequencies are found for the A_2u_ mode^49^ and thus more expensive hybrid functional calculations are required to represent the electronic and atomic structure correctly. All results presented herein were obtained using the HSE06 hybrid functional^50^ with 25% Hartree–Fock exchange and an *ω* parameter of 0.11. HSE06 obeys the generalised Koopman's theorem, which means it yields improved vertical excitation energies, charge trapping energies and charge transition levels.^51^ In order to reduce the computational cost of the hybrid functional calculations, the auxiliary density matrix method (ADMM)^52^ was used. It employs a reduced basis set for the Hartree–Fock exchange calculation and thus allows the computation of larger cells, which would otherwise be prohibitively expensive in hybrid DFT.

Defect formation energies (DFE) were calculated using:1
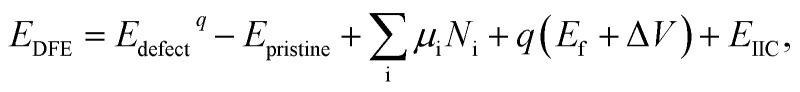
where *q* is the charge state of the defect, *μ*_i_ is the chemical potential of species i, *N*_i_ is the number of removed atoms of species i and *E*_f_ is the Fermi level of the pristine system. Δ*V* is a potential alignment term, and *E*_IIC_ is an image interaction correction (IIC). Both Δ*V* and *E*_IIC_ are finite size dependent terms, which arise from periodic boundary conditions and tend to zero with increasing simulation box size. The method proposed by Lany and Zunger was used to account for image interaction corrections.^53^ Potential alignment is a volume dependent term, which arises from the convention of setting the average electrostatic potential (〈*V*〉) to zero in periodic DFT calculations. Thus, 〈*V*_defect_〉 ≠ 〈*V*_pristine_〉 and the electrostatic potential of pristine bulk material is not recovered far away from the defect. This shift in energy needs to be accounted for by aligning the average electrostatic potential of the vacuum region in the defective and pristine simulation cells.^54^ For the surface calculations, a vacuum gap of 20 Å ensured that the interaction between the surface and its periodic image tends to zero and thus allows for accurate potential alignment.

The surface slab consisted of a 2 × 4 × 8 TiO_2_ cell (384 atoms), unless stated otherwise. Reaction barriers were calculated using climbing image nudged elastic band calculations.^55^

As discussed below, both O vacancies and Ti interstitials induce electron polaron states localized on Ti ions at low temperatures. At high temperatures of surface reduction electrons trapped at these polaron states are released into the conduction band.^56^ These effects are not included in our static periodic DFT simulations at 0 K are subject of further studies.

## 3 Ti_i_ at the (110) surface

To determine how the Ti_i_ formation energy depends on the distance from the surface, the defect was calculated at various positions in an eight layer (110) surface terminated slab. Table 1 lists these DFEs as well as the value calculated for an interstitial in bulk TiO_2_. The chemical potentials of O (*μ*_O_) and Ti (*μ*_Ti_) were chosen to refer to conditions during an annealing process (1000 K and 10^−6^ atm O_2_ pressure), as outlined in ref. 57, and all atoms in the slab were allowed to relax.

**Table 1:** DFE for a Ti_i_ at various depths below the surface of an 8 layer TiO_2_ slab

Layer	DFE/eV
First	3.51
Second	3.01
Third	3.15
Fourth	3.12
Bulk	3.02

As can be seen in Table 1 that for a Ti_i_ in the first bilayer (*i.e.* between the surface plane and subsurface plane of Ti atoms) the DFE is almost 0.5 eV higher than for a bulk interstitial. This is related to the surface relaxation, where the top plane of atoms relaxes downwards upon creation of the (110) surface. Thus the two planes of atoms are closer together leading to bigger distortion upon Ti_i_ incorporation and making it less energetically favourable. An interstitial in the next layer down, however, is energetically more favourable with a DFE of 3.01 eV, which is close to the bulk value of 3.02 eV. As the interstitial is moved inside the slab, the bulk DFE should be recovered. However, the computational cost limits the number of layers which can be computed, which means that the middle layers do not fully recover bulk behaviour, as previously reported for the calculation of the surface energy.^58^ Nevertheless, the trend shows that subsurface interstitials in the second layer are more stable than these in the top and third layer. This can have implications for interstitial indiffusion, where a higher concentration of interstitials sits close to the surface and can contribute to surface reactions.

**Fig. 1:**
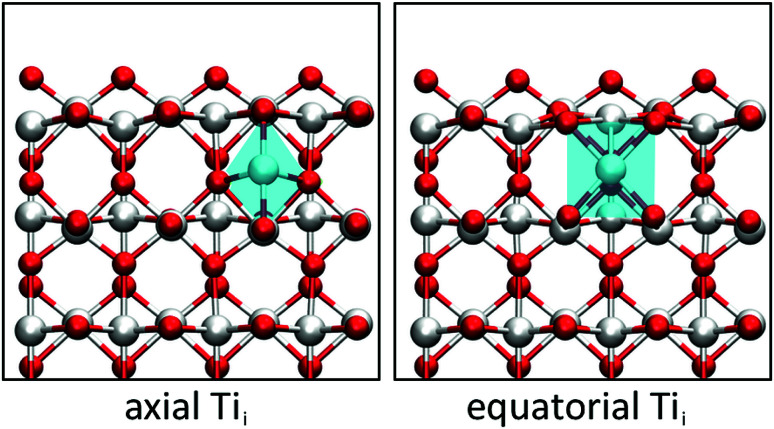
Axial and equatorial Ti_i_ at the (110) surface of rutile TiO_2_. The blue surface represents the plane cut through the TiO_6_ octahedron showing the elongated axial diamond base for axial Ti_i_ and the square base for equatorial Ti_i_. (Red = O, white = Ti).

A closer look at the geometry of the subsurface Ti_i_ reveals that two configurations are possible. The Ti_i_ can either bond to one surface O atom (axial configuration) or to two surface O atoms (equatorial configuration). The two configurations differ in a 90° rotation of the distorted octahedron of the Ti_i_ and a translation by half a lattice vector along [001], as shown in Fig. 1. Both configurations adopt a triplet Ti_i_^3+^ state with four localised polarons in the vicinity, though the axial configuration is 0.24 eV higher in energy than the equatorial configuration. The difference can be traced down to the geometry of a Ti_i_, which sits in an elongated octahedron. Since the distance between the top and second layer is reduced upon creating the (110) surface, the octahedron preferentially orientates the longer axis parallel to the surface plane instead of along the surface normal.

A bulk Ti_i_ induces four polaronic states, which sit at 1.12 eV below the conduction band minimum (CBM), which is in reasonable agreement with infrared adsorption measurements showing a peak at 1.18 eV.^59^ Two polarons localise on lattice Ti atoms and two localise on the interstitial and a neighbouring lattice Ti site jointly, in agreement with a previous report.^51^

**Fig. 2:**
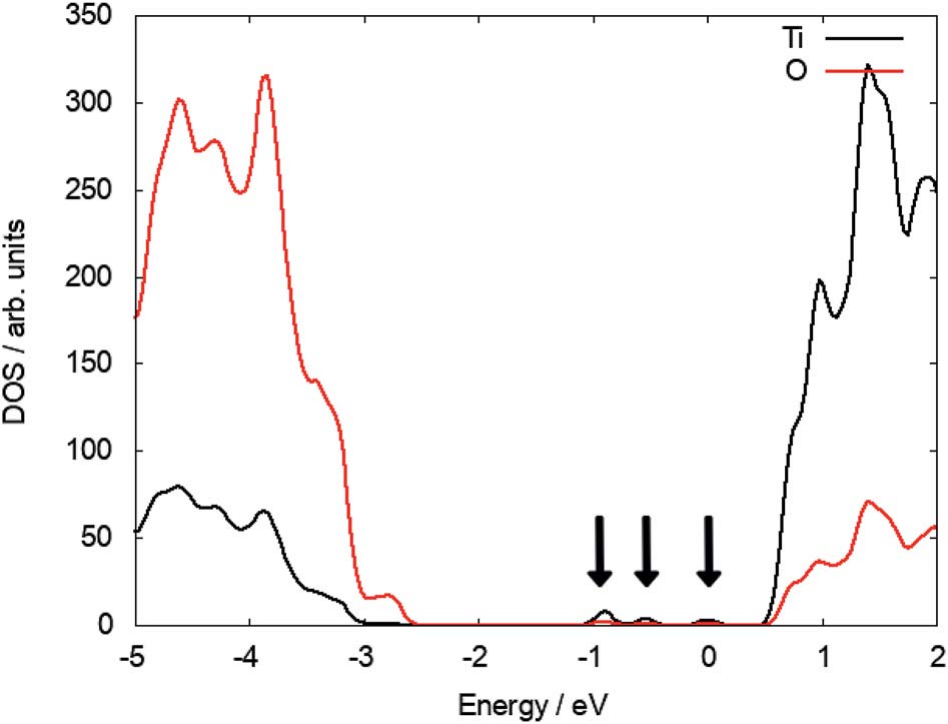
DOS plot of a subsurface Ti_i_ illustrating the polaronic states in the bandgap of TiO_2_.

At the (110) surface two polarons localise on surface Ti^5f^ sites (the five coordinated Ti atom at the surface of TiO_2_) and two on subsurface sites, which can contribute to the reactivity of the (110) surface. Since the surface breaks the lattice symmetry, the polaron states are not degenerate and located at different positions in the bandgap. Three states can be identified at 0.67 eV, 1.18 eV and 1.57 eV below the CBM (see Fig. 2). As the interstitial is moved inside the slab, these states move closer in energy and ultimately become degenerate and recover the polaron state of Ti_i_ in the bulk (see S3 in ESI†).

## 4 Surface oxygen vacancies

As a TiO_2_ crystal is annealed at high temperatures in a reducing atmosphere, oxygen vacancies are created at the surface. A bridging oxygen vacancy was calculated to have a DFE of 1.67 eV, which is lower than the DFE of a bulk vacancy at 2.54 eV (both calculated at 1000 K and 10^−6^ atm O_2_ gas pressure as illustrated in S2 in ESI†). The reduction in DFE is due to the under-coordinated nature of the bridging oxygen, which forms only two bonds instead of three with neighbouring Ti atoms and the DFE is approximately 2/3 of the bulk value. Furthermore, creating two neutral oxygen vacancies adjacent to one another has a DFE of 3.21 eV, indicating that this is marginally favourable compared to two isolated neutral vacancies. Indeed some clustering of v_O_ has been observed experimentally.^19,60^ While whether the vacancies are being formed next to each other or coalesce while diffusing on the surface is unclear, experimental images of highly reduced (110) surfaces show strands of missing bridging oxygen and surface Ti atoms. Investigating oxygen vacancy diffusion and mechanisms for oxygen defect-complex formation is beyond the scope of this paper and will motivate future work.

The neutral v_O_ adopts a triplet state, where the two unpaired electrons form polaron states in the bandgap of TiO_2_. The two states lie at 0.9 eV and 1.15 eV below the CBM, which correspond to a polaron on a surface Ti next to the O vacancy and a polaron on a Ti lattice atom in the subsurface layer, respectively. The polaron states are shifted compared to a bulk v_O_, which induces two polarons at 0.8 eV and 1.31 eV below the CBM, respectively.

## 5 Surface to bulk diffusion

The DFEs for a Ti_i_ show a favorable trend towards the bulk of rutile TiO_2_. The proposed process for indiffusion is as follows: surface oxygen is removed during annealing, leading to Ti_i_ formation at the surface. Due to the concentration gradient, Ti_i_ atoms diffuse into the bulk given the diffusion barrier can be overcome at sufficiently high temperatures, leading to an overall reduction of the crystal.

**Fig. 3:**
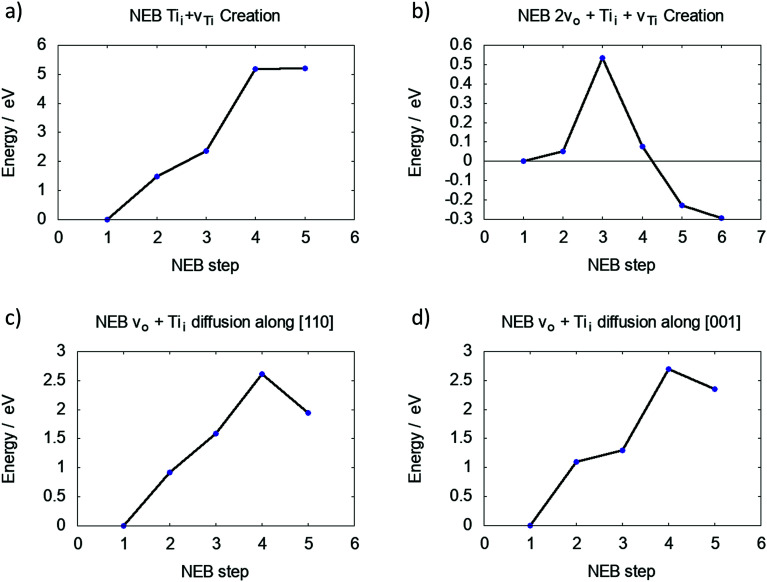
Barriers for (a) moving Ti^5f^ into an interstitial position, (b) moving Ti^5f^ into an interstitial position with two neighbouring v_O_, (c) Ti_i_ diffusion away from v_Ti_ along [110] direction and (d) Ti_i_ diffusion away from v_Ti_ along [001] direction.

The barrier for creating a v_Ti_ and Ti_i_ between the first and second Ti layers is over 5 eV, as shown in Fig. 3a. This is lower than 6.64 eV calculated for the two individual defects in bulk rutile TiO_2_. However, the final state is not an energy minimum and the interstitial Ti will relax back into the vacancy site.

The same process was investigated for a reduced surface. The barrier is only slightly lowered for a surface containing a single v_O_ (4.93 eV and no energetic minimum), yet significantly lowered for a surface with two adjacent v_O_ (see S4 in ESI†), which is shown in Fig. 3b. Two neighbouring O vacancies were created at O_Br_ sites exposing a four-fold coordinated Ti atom. The barrier to move this Ti atom into an interstitial position is 0.54 eV and the final interstitial site is an energy minimum, which is 0.29 eV lower than the energy of the Ti^4f^ (four coordinated Ti atom at the surface of TiO_2_) atom in its lattice site. Effectively a Schottky defect was formed, where the Ti atom maximises its coordination with lattice O atoms by adopting an interstitial geometry (see ESI†).

Fig. 3c and d illustrate the diffusion barriers along [110] and [001] for this Ti_i_ at a reduced (110) surface (2v_O_). Both barriers are approximately 2.5 eV, much higher than the bulk diffusion barrier along these crystallographic directions (see S6 in ESI†), which can be attributed to the interaction of Ti_i_ with the defect complex. An interstitial in pristine TiO_2_ binds only weakly to the lattice O atoms, however, the Ti_i_ next to a Schottky defect binds strongly to the under-coordinated lattice O atoms, making their separation more energetically costly.

The subsurface interstitial site is even more stable for three bridging oxygen vacancies in a row. In that case a Ti atom in an interstitial site next to a Ti vacancy is 0.35 eV lower in energy than the Ti atom in the lattice site, indicating that further reducing the surface favours the creation of subsurface Ti interstitials. Previously it has been reported that an under-coordinated Ti atom will spontaneously relax into the interstitial site,^19^ however, this could not be reproduced, indicating the presence of a small barrier for the lattice Ti atom to move into the adjacent subsurface interstitial site.

**Fig. 4:**
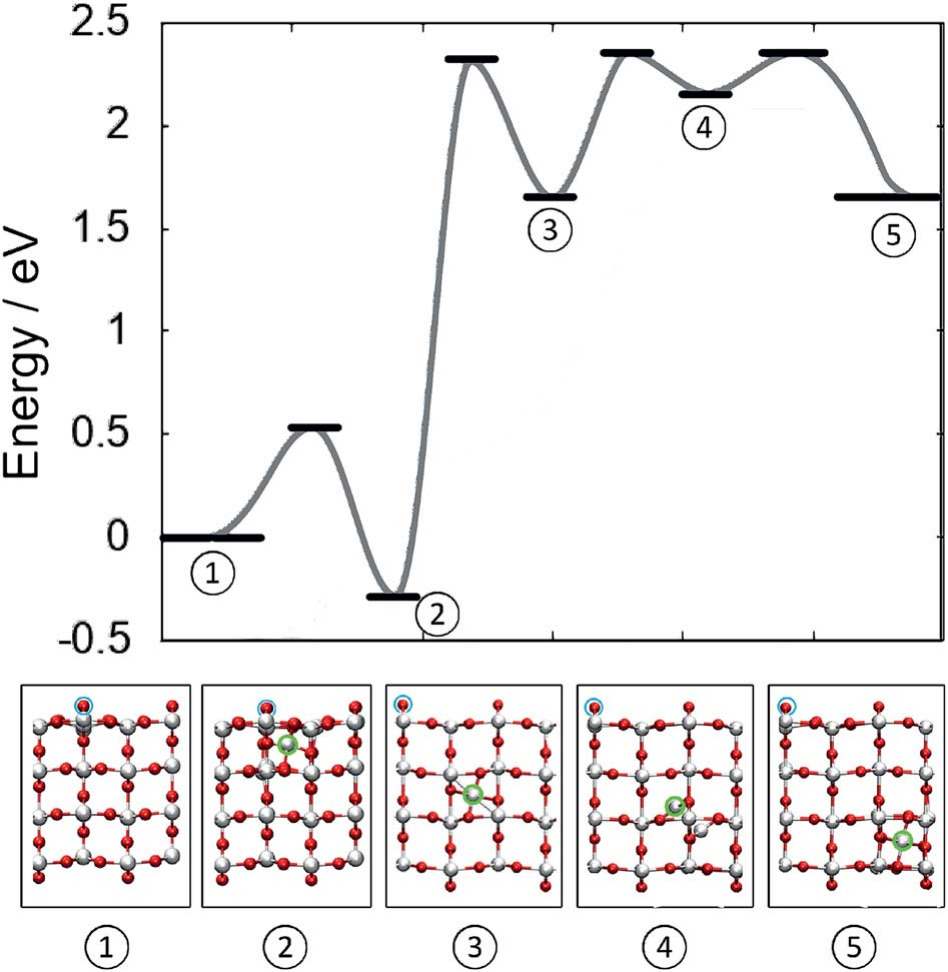
Schematic showing the barriers for the creation and indiffusion of a Ti_i_ from a reduced (110) surface into the bulk. Zero energy refers to a surface with two v_O_ at neighbouring bridging oxygen sites and no Ti_i_. Atomic structures of the energetic minima are illustrated below. Cyan circles indicate the location of the two v_O_ on the surface of TiO_2_. (Red = O, white = Ti).

Fig. 4 illustrates the complete potential energy path for dissociation of a 4-coordinated surface Ti ion from surface O di-vacancy in the direction of TiO_2_ bulk. The separation of Ti_i_ from the defect complex constitutes the rate determining barrier, after which the diffusion barrier almost recovers bulk diffusion. From configuration (3) to (5) (see labels in Fig. 4) the barrier is 0.7 eV, which is 0.2 eV higher than the corresponding barrier in the bulk and the transition structure (4) is 0.3 eV higher than the split interstitial in bulk TiO_2_. These results confirm that the surface reduction can facilitate the formation of stable Ti_i_ in the bulk of rutile TiO_2_.

## 6 Discussion and conclusion

The existence of near surface Ti_i_ has been linked to the chemical activity of the (110) surface of rutile TiO_2_,^61,62^ but the behaviour of these defects is still poorly understood. In bulk rutile TiO_2_, there exists only one possible interstitial site for Ti atom. Such an interstitial retains one of its four valence electrons and induces three small polarons on lattice Ti sites, thus creating four Ti^3+^ species. An interstitial at the surface, however, can adopt one of the two possible configurations, termed axial or equatorial geometry. The polarons associated with these subsurface Ti_i_ sit on surface and subsurface Ti lattice sites. Due to the difference in screening of these trapped charges on the surface, the polaron states are not degenerate and are located between 0.67 eV and 1.57 eV below the CBM and thus may impact the chemical reactivity of the surface. This is consistent with an increased photo-absorption in the visible spectrum for reduced TiO_2_ crystals.^24^

As the interstitial moves into the bulk, the polaron states move closer in energy and converge at 1.02 eV below the CBM for an interstitial in the fourth layer, which is slightly higher than the state at 1.12 eV below the CBM for a bulk Ti interstitial. Electron energy loss spectroscopy (EELS) has revealed a bandgap feature, which is reported at either ∼1 eV (ref. 63 and 64) or 0.8 eV,^59,65,66^ and attributed to polaron states below the CBM of TiO_2_. Bulk oxygen vacancies agree well with the shallow polaron model (see ESI†), whereas both bulk Ti_i_ and v_O_ defects induce a state in agreement with the deeper polaron state. The discrepancy in the reported experimental values may be traced to sample treatment and preparation methods, creating oxygen vacancies or near surface Ti interstitials. The bulk polaron states sit at different energies to the surface polarons giving rise to a multitude of signals in the range of ∼0.8–1.0 eV below the CBM.^25–27,59^

The barrier to create a Ti_i_ at the fully oxidised (110) surface was calculated at >5 eV. However, this barrier is reduced to just ∼0.5 eV for a Ti atom next to two bridging oxygen vacancies. In this configuration the energy of the interstitial site is lower than that of the Ti atom at its lattice site. It has long been proposed that such a mechanism should exist,^32^ and our results provide strong support to this assertion.

To further separate the neutral Ti_i_ from the surface tri-vacancy, ∼2.5 eV is required, both for [110] and [001] diffusion. At high temperatures, polarons are mobile and electrons can become delocalised.^31^ Therefore Ti_i_ can be locally charged when performing diffusion hops. The calculations for bulk diffusion indicate that the barrier is lower for Ti_i_^4+^ diffusion. Since accurate surface charge corrections are still challenging to calculate, only the neutral case was considered for the subsurface Ti interstitial, however, it can be expected that Ti^4+^ ion diffusion will have a lower barrier following the same trend as a bulk interstitial.

The calculated barrier to separate the interstitial from the defect complex is comparable to the barrier of 3.3 eV, which was previously calculated for Ti_i_ dissociation from extended defects using a micro-kinetic model.^43^ Furthermore, data from tracer self-diffusion suggests an activation energy of 2.47 eV for Ti_i_ migration in reduced TiO_2_ crystals^44^ and estimates from scanning probe measurements report a barrier of ∼3 eV for Ti to migrate from the surface into the bulk, all in good agreement with our results.

However, the reverse barrier is much lower at 0.67 eV along [110] and 0.35 eV along [001]. Therefore, reoxidation of TiO_2_ should be much faster than reduction *via* heat treatment. This is in agreement with experimental procedures, where rutile TiO_2_ crystals are annealed at 823 K for one hour, producing many subsurface Ti interstitials but the signal from bulk Ti^3+^ indicates very low concentrations. However, oxygen exposure at the same temperature will lead to rapid reoxidation of the surface; drawing Ti_i_ out of the near-surface region to form new TiO_2_ islands and ultimately a pristine (110) surface can be obtained in just minutes, as observed in STM measurements.^16,32,38^

After the interstitial has been separated from the defect complex on the surface, the diffusion barrier almost recovers the bulk diffusion value. This is due to the effective screening in TiO_2_, which has a high dielectric constant of ∼100. Therefore, the indiffusion of Ti is limited by the barrier separating the interstitial from the v_Ti_ + 2v_O_ complex. Furthermore, it is postulated that the defect cluster will grow along the [001] direction, as the lattice atoms next to the defect cluster are less tightly bound. Such a process would explain the observed streaks in STM measurements^39^ and could be related to the formation of the 1 × 2 reconstruction of the (110) surface. However, further theoretical work is required to confirm this proposed mechanism.

To conclude, our results demonstrate that creation of oxygen di-vacancies at bridging oxygen sites at the TiO_2_ rutile (110) surface facilitates formation Ti_i_ at the surface. The barrier to separate the interstitial Ti ion from the surface oxygen vacancies is ∼2.5 eV. However, the bulk diffusion barrier is recovered after the interstitial is moved away from the vacancy complex. These results shed light on the atomistic mechanism of Ti_i_ formation and contribute to our understanding of the TiO_2_ surface reduction and reoxidation.

## Conflicts of interest

There are no conflicts to declare.

## Supplementary Material

RA-009-C9RA01015G-s001
