# Supplementary material for: The role of surface reduction in the formation of Ti interstitials
Source: RSC Adv. 2019 Apr 17;9(22):12182–8. doi: 10.1039/c9ra01015g (PMC9063666; doi:10.1039/c9ra01015g)
Supplement: RA-009-C9RA01015G-s001 [file RA-009-C9RA01015G-s001.pdf]

Electronic Supplementary Material (ESI) for RSC Advances.
This journal is © The Royal Society of Chemistry 2019

Supplementary Information: The Role of Surface Reduction in the Formation of Ti Interstitials

Julian Gaberle, Alexander Shluger

March 9, 2019

1 ESI

The chosen method was verified by comparing the bulk electronic and geometric structure as well as the (110) surface structure to experimental results and theoretical calculations reported in literature. The optimised lattice vectors were found to be a = 4.60 Å and c = 2.93 Å, which is in good agreement with a = 4.59 Å and c = 2.96 Å found from neutron powder diffraction experiments.[1]

Each Ti atom is six fold coordinated, while every O atom is three fold coordinated. The Ti-O bonds can be separated in two types, axial and equatorial. Each Ti atom has two axial Ti-O bonds at an angle of 180° wrt. each other and four equatorial bonds, at 90° wrt. each other. The optimised bulk bond lengths are 1.99 Å for axial and 1.94 Å for equatorial Ti-O bonds, which is in good agreement with experimentally measured bond lengths (see Table S1). A direct bandgap of 3.5 eV at the Gamma-point is slightly larger than the experimentally measured value of 3.03 eV[2,3], but compares well with reported values from DFT calculations using the same functional[4] and from full frequency G_0W_0 calculations.[5] The top of the valence band (VBM) is composed mostly of O 2p character, whereas the bottom of the conduction band (CBM) has predominantly Ti 3d character. Bader charges on Ti are +2.4e and on O -1.2e, respectively, indicative for ionic bonding. The heat of formation of a unit of TiO_2 was calculated at -9.15 eV, which is in agreement with the experimental value of -9.72 eV.[6]

The ability of our model to represent the (110) surface structure accurately was also verified. The (110) surface is the lowest energy rutile surface and has been studied extensively.[7–10] It consists of alternating corner sharing TiO_6 and TiO_5 octahedra, where the Ti atom is located in the centre of the octahedron. The Ti atoms lie in planes parallel to the surface plane with one oxygen per unit of TiO_2 sticking out of the plane, as illustrated in Fig. S1(right).

Previously theoretical studies show, that surface properties, such as surface energy or adsorption energies of small molecules, converge slowly with increasing number of TiO_2 layers in the simulated slab.[11,12] Methods to improve convergence include H termination of dangling bonds on the bottom surface or constraining the bottom two layers of the slab in their bulk geometry. However, H termination can lead to artificial dipoles in the system and thus can influence the electronic structure, which is particularly detrimental to shallow polaronic states. Bulk termination is useful for adsorption calculations, which primarily rely on accurate representation of the geometric and electronic structure of the surface and rely less on an accurate description of the bulk properties within the slab.

However, in this work adsorption as well as substitution processes are investigated, which require a thick TiO_2 slab to be modelled, in order to get accurate bulk properties in the middle of the slab, as well as a correctly represented surface. Fig. S1 shows the convergence of surface energy with number of TiO_2 layers in the slab. The typical even-odd layer oscillations are not seen since only even layer slabs were calculated as they were found to converge even more slowly.[13] As expected convergence is slow and could not be reached up to a 10 layer system. However, the obtained value of about 0.6 eV per surface unit cell in a 10 layer slab compares well with published results.[11–13] As the computational cost increases drastically with the number of atoms modelled, a balance between accuracy and computational cost has to be achieved. Thus, an eight layer slab was chosen for this work, in which all atom positions are optimised. A 20 Å vacuum gap was found sufficient to minimise periodic interactions between slabs in the out of plane direction.

The slow convergence of surface energies, adsorption energies and other surface related properties can be traced back to atomic relaxations upon creating the surface, as illustrated on the right side in Fig. S1. When the TiO_2 crystal is cleaved to create a (110) surface, undercoordinated Ti and O atoms are produced. These undercoordinated atoms show the largest surface relaxations from their

Atom Pair	Bulk Bond Length	Exp. Bond Length	This work	% difference
Ti(1)-O(1)	1.94	1.71	1.81	5.9
Ti(1)-O(2)	1.99	2.15	2.03	-5.6
Ti(1)-O(3)	1.94	1.99	2.09	5.0
Ti(2)-O(2)	1.94	1.84	1.94	5.4
Ti(2)-O(4)	1.99	1.84	1.80	-2.2
Ti(3)-O(4)	1.94	2.00	2.03	1.5
Ti(3)-O(5)	1.99	1.92	1.92	0.0
Ti(3)-O(6)	1.94	1.94	1.87	-3.6
Ti(4)-O(3)	1.99	1.97	1.83	-7.1
Ti(4)-O(5)	1.94	1.99	1.99	0.0
Ti(4)-O(7)	1.99	2.18	2.19	0.5

Table S1 Bond lengths in TiO_2, illustrating atomic relaxations on the (110) rutile surface. Experimental values have been adapted from[14], which were obtained from surface X-ray diffraction measurements. The percentage change corresponds to the experimental value compared to the theoretical value. All bond lengths are given in Å and labeled according to Fig. S1.

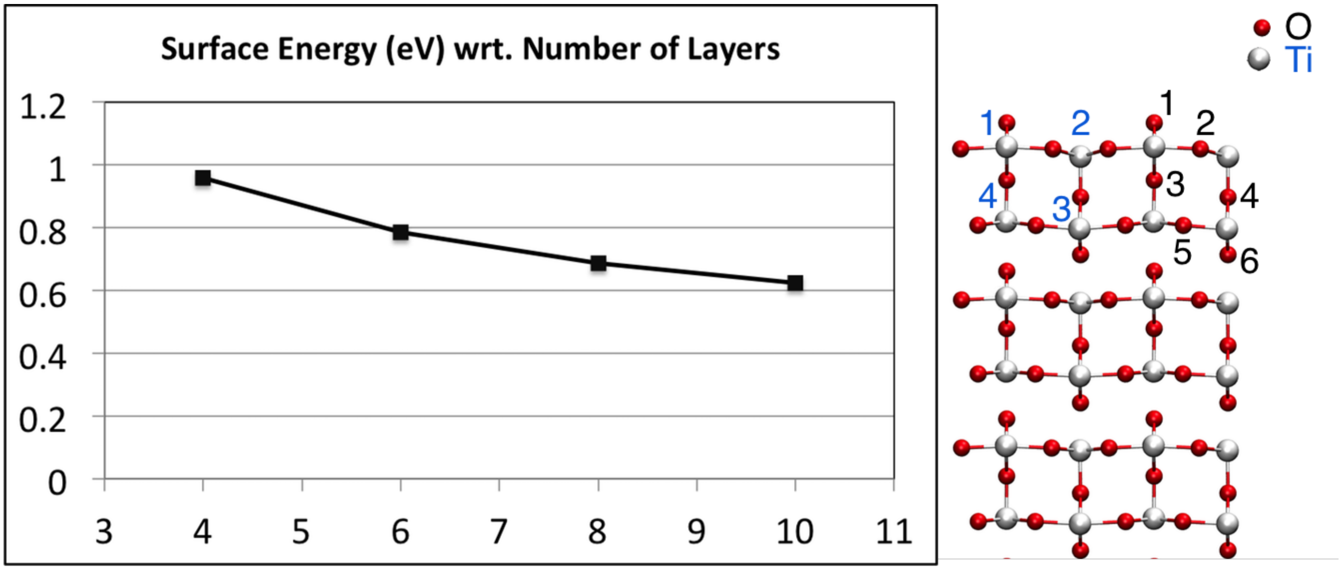

Figure S1 Left: Plot of surface energy versus number of layers in the 2x4 (110) surface slab of rutile TiO_2. Only even layer slabs were calculated to check convergence. Right: Six layer surface slab illustrating atomic surface relaxations.

bulk lattice sites (see Table S1). The O(1) is termed bridging oxygen (O_{Br}) and relaxes into the surface shortening the Ti-O bonds (1.81 Å). Similarly, the five fold coordinated surface Ti atom relaxes inwards, elongating the in-plane Ti-O bonds and compressing the Ti-O bond along the surface normal. The top layer of atoms displays the largest relaxations, which is consistent between theory and experiment, albeit the magnitude of relaxations varies by up to ~6%. Interestingly, both experiment and theory indicate the formation of bi-layers. While the distance between the first and the second layer decreases, the distance between the second and third layer increases. This bi-layer formation leads to slow convergence of the out-of-plane bonds with increasing number of layers in the slab, while in-plane bonds converge more rapidly. In Fig. S1 the bi-layer formation is illustrated by omitting Ti-O bonds between the bi-layers. Overall, the agreement between our data and experimental data is largely within experimental error of about 0.1 Å.

2 Point defects

Figure S2 shows a defect formation energy diagram for a Ti interstitial, Ti vacancy, O interstitial and O vacancy in bulk rutile TiO_2. It can be seen that the +4 interstitial and a +2 vacancy have the lowest DFEs at a wide range of Fermi level positions.

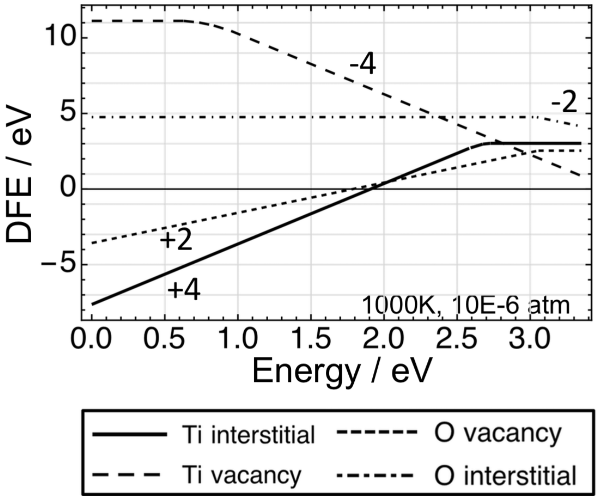

Figure S2 Defect formation energy diagram for intrinsic point defects in bulk rutile TiO_2. The respective charge state of the defects has been indicated and all DFEs have been corrected for mirror charge interactions across the periodic boundary (Lany-Zunger corrections were used.[15])

Figure S3 illustrates the DOS of a Ti_i in various layers from the (110) surface of rutile TiO_2. The bandgap states induced by the defect become more degenerate as the interstitial is moved into the slab towards the bulk. of rutile TiO_2.

Figure S4 illustrates the atomic arrangement of a Ti interstitial next to a defect complex comprising of two bridging oxygen vacancies and an adjacent Ti vacancy, forming essentially a surface Schottky defect.

Figure S5 illustrates the density of states of a bulk Ti interstitial and a bulk O vacancy. The polaronic Kohn-Sham states in the bandgap can clearly be identified.

2.1 Ti Interstitial Diffusion

In order to better understand the mobility of the interstitial Ti atoms, NEB calculations determining diffusion paths were performed. There are two possible low energy diffusion paths for a Ti_i defect: along the (001) direction, which corresponds to diffusion through a hollow channel in the TiO_2 lattice, and via an interstitialcy mechanism along the (110) direction.

Fig. S6 illustrates the barriers for diffusion along the (001) and (110) crystallographic directions. For a neutral Ti interstitial (Ti_i^{+3}+3 polarons) the diffusion barrier along the (001) crystallographic direction is 0.34 eV, which is lower than previously reported values obtained with a GGA functional.[16,17] Due to the lattice symmetry, the diffusion path has length $c/2$ for which the barrier is shown (see insets of atomic configurations).

The interstitialcy mechanism is the lowest in energy for diffusion along the (110) crystallographic direction. The calculated path displays a two step process with barriers 0.5 eV and 0.55 eV. Due to lattice symmetry, these two barriers should be equal and the discrepancy is attributed to not fully relaxed transition structures. A local minimum is found for a Ti_i-v_{Ti}-Ti_i complex, which is 0.23 eV higher in energy than the Ti_i configuration in a rutile lattice. Interestingly, previous reports show the intermediate structure to be lower in energy than the start and end configurations.[16]

The diffusion of a Ti_i^{+4} defect was also calculated, which has respective barriers of 0.25 eV along (001) and 0.31 eV along (110), which is close to reported values of 0.31 eV along (001) and 0.23 eV along (110).[16] During the diffusion process the electron which is localised on Ti_i leaves the interstitial to form a polaron on a nearby lattice Ti atom. The charged interstitial and the polaron states will attract each other, increasing the activation energy for diffusion. GGA barriers are lower, partly since the electrons of the interstitial are delocalised in the conduction band.

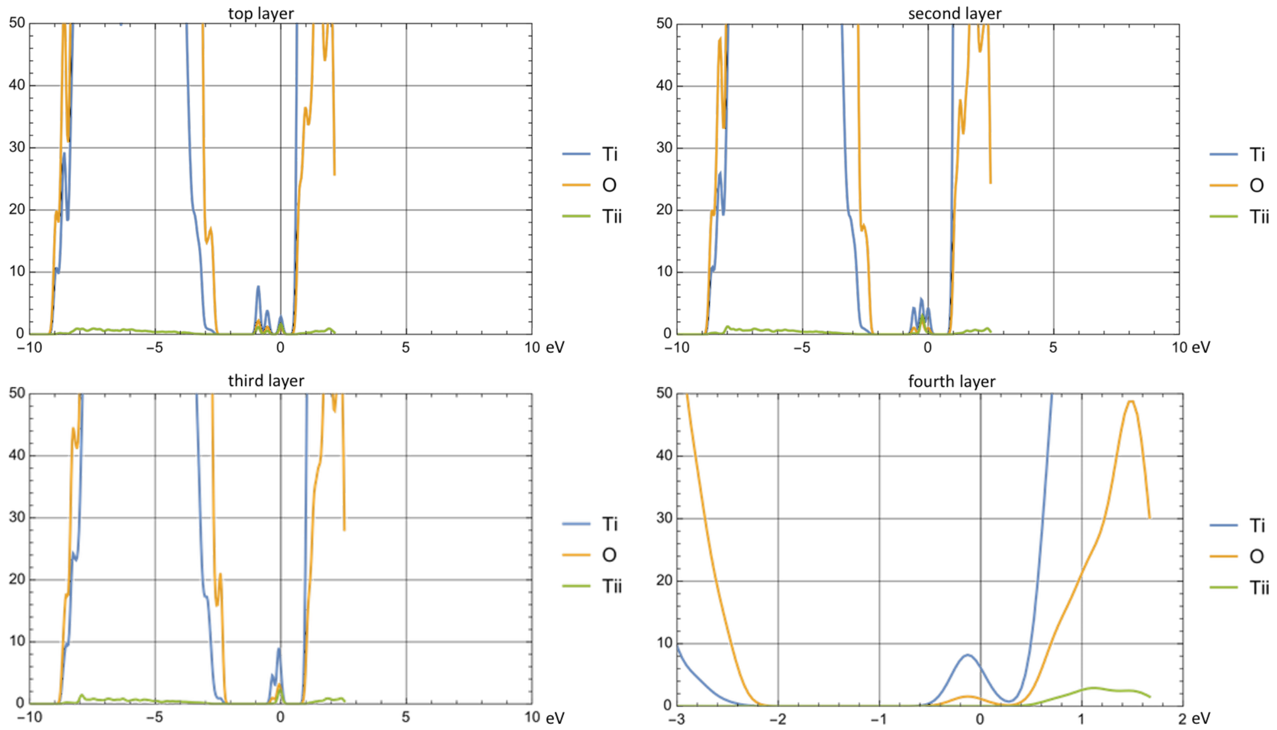

Figure S3 DOS of a Ti_i in the top, second, third and fourth surface layer in rutile TiO_2 at the (110) surface.

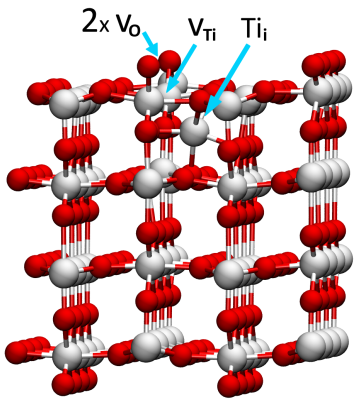

Figure S4 Atomic structure of a subsurface Ti_i next to a surface Schottky defect ($2\times v_O$+Ti_i)

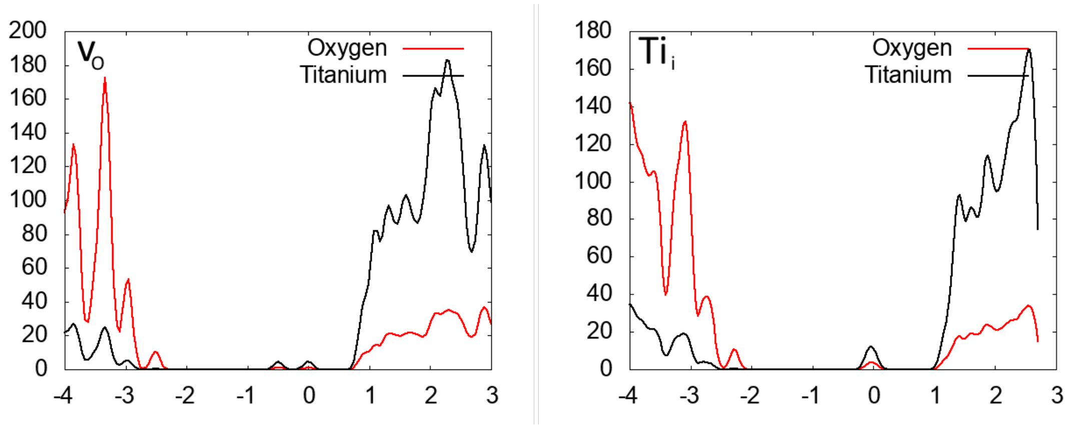

Figure S5 Density of states for v_O and Ti_i defects in bulk rutile TiO_2. The x-axis is set to zero at the highest occupied Kohn-Sham state.

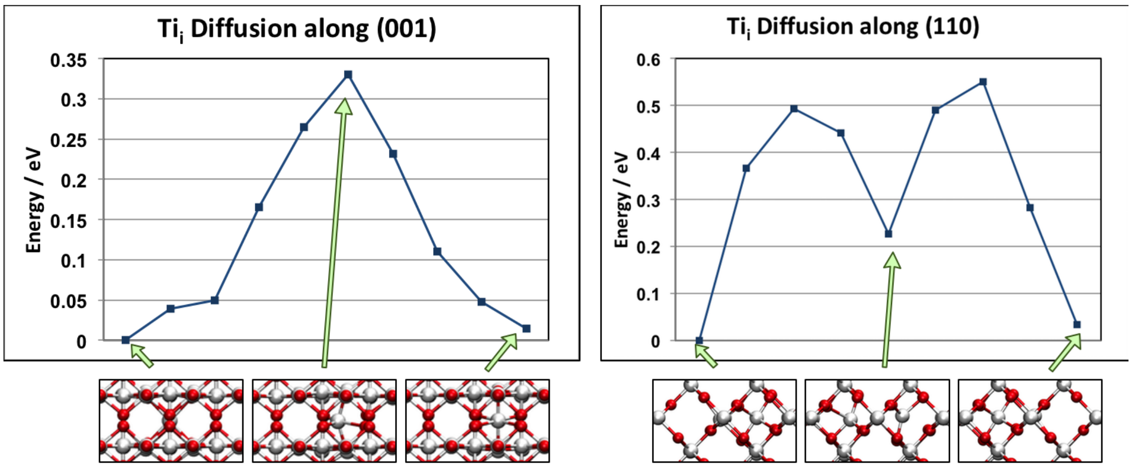

Figure S6 Barrier for Ti_i diffusion along the (001) and (110) crystallographic directions in bulk TiO_2. The insets illustrate atomic geometries of three points along the diffusion path as indicated by the arrows. (Red = O, white = Ti)

References

[1] C. J. Howard, T. M. Sabine and F. Dickson, *Acta Crystallographica Section B Structural Science*, 1991, **47**, 462–468.

[2] Y. Tezuka, S. Shin, T. Ishii, T. Ejima, S. Suzuki and S. Sato, *Journal of the Physical Society of Japan*, 1994, **63**, 347–357.

[3] J. Pascual, J. Camassel and H. Mathieu, *Physical Review B*, 1978, **18**, 5606–5614.

[4] P. Deák, B. Aradi and T. Frauenheim, *Physical Review B - Condensed Matter and Materials Physics*, 2011, **83**, 1–7.

[5] W. Kang and M. S. Hybertsen, *Phys. Rev. B*, 2010, **82**, 085203.

[6] J. Cox, D. Wagman and V. Mendvedev, *Hemisphere Publishing Corp., New York*, 1989.

[7] S. Wendt, R. Schaub, J. Matthiesen, E. Vestergaard, E. Wahlström, M. Rasmussen, P. Thostrup, L. Molina, E. Lægsgaard, I. Stensgaard, B. Hammer and F. Besenbacher, *Surface Science*, 2005, **598**, 226–245.

[8] U. Diebold, J. Lehman, T. Mahmoud, M. Kuhn, G. Leonardelli, W. Hebenstreit, M. Schmid and P. Varga, *Surface Science*, 1998, **411**, 137–153.

[9] S. J. Thompson and S. P. Lewis, *Physical Review B - Condensed Matter and Materials Physics*, 2006, **73**, 2–5.

[10] C. L. Pang, R. Lindsay and G. Thornton, *Chemical Reviews*, 2013, **113**, 3887–3948.

[11] Z. Helali, A. Markovits, C. Minot, A. Dhouib and M. Abderrabba, *Chemical Physics Letters*, 2012, **531**, 90–93.

[12] T. Bredow, L. Giordano, F. Cinquini and G. Pacchioni, *Physical Review B - Condensed Matter and Materials Physics*, 2004, **70**, 1–6.

[13] P. M. Kowalski, B. Meyer and D. Marx, *Physical Review B*, 2009, **79**, 115410.

[14] G. Charlton, P. B. Howes, C. L. Nicklin, P. Steadman, J. S. G. Taylor, C. A. Muryn, S. P. Harte, J. Mercer, R. McGrath, D. Norman, T. S. Turner and G. Thornton, *Physical Review Letters*, 1997, **78**, 495–498.

[15] S. Lany and A. Zunger, *Phys. Rev. B*, 2008, **78**, 235104.

[16] H. Iddir, S. ÖÇğüt, P. Zapol and N. D. Browning, *Physical Review B - Condensed Matter and Materials Physics*, 2007, **75**, 2–5.

[17] A. M. Asaduzzaman and P. Krüger, *Journal of Physical Chemistry C*, 2010, **114**, 19649–19652.
